# Supplementary material for: Ribosomal RNA methylation by GidB modulates discrimination of mischarged tRNA
Source: bioRxiv. 2026 Apr 23:2021.03.02.433644. Preprint. [Version 5] doi: 10.1101/2021.03.02.433644 (PMC13131534; doi:10.1101/2021.03.02.433644)
Supplement: Supplement 1 [file media-1.pdf]

Supplementary information-Figures

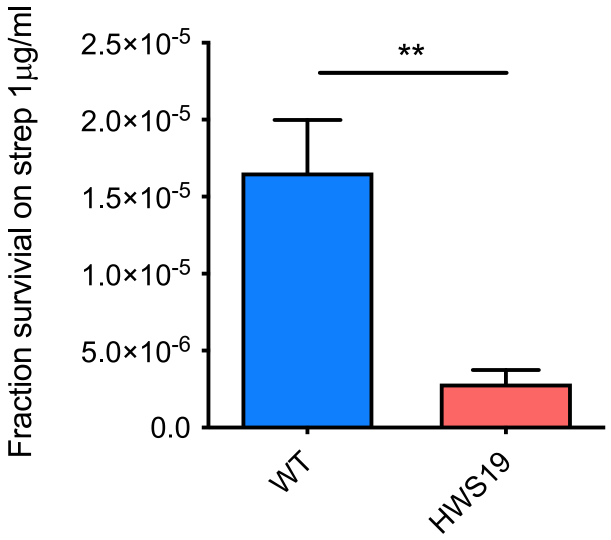

Figure S1. Susceptibility to streptomycin of WT and HWS19. HWS19 is more susceptible to MIC of streptomycin than WT. Data are presented as means of three biological replicates ± SD. **P<0.01

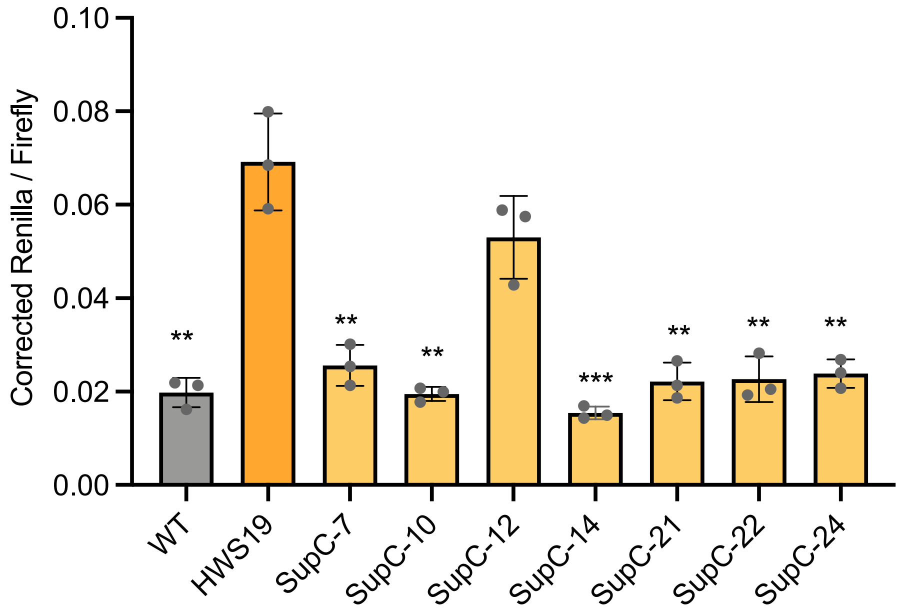

Figure S2. Mistranslation level of the other suppressor candidates with *gidB* mutations. N to D mistranslation rates were measured in 7 suppressor candidates compared with wild type and HWS19 by Renilla-Firefly dual luciferase reporter. Mutations in each suppressor candidates were shown in Table S2.

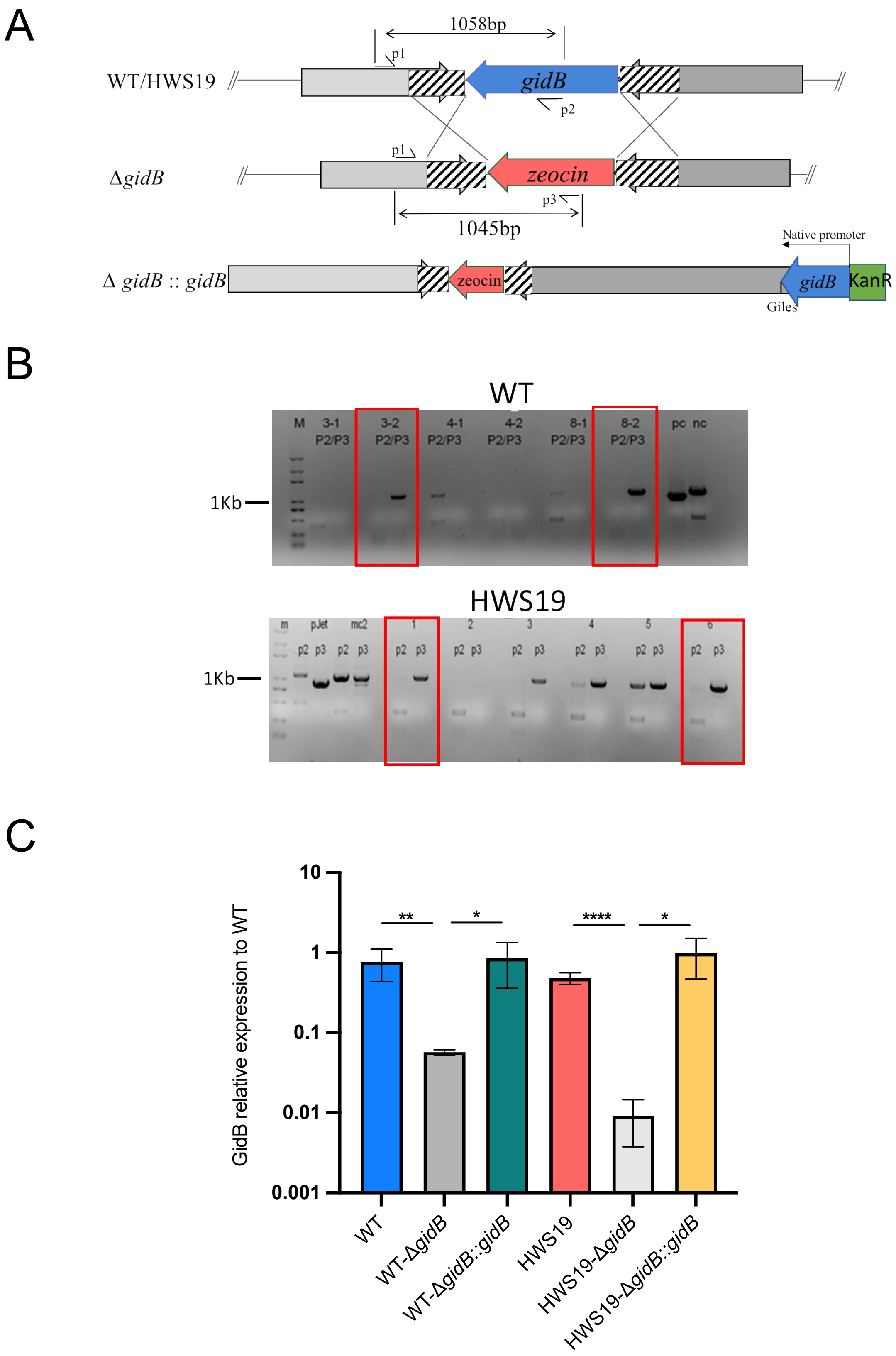

Figure S3. *gidB* deletion and complementation in WT and HWS.19. A.Homologous recombination was performed for deletion of *gidB,* replaced with zeocin resistance marker*. gidB* complement was integrated into Giles site with pml1357. B. PCR screening to verify successfully deletion of *gidB* in both wildtype background and HWS19 backgroud. Primer 1 (P1) is upstream of *gidB*, primer 2 (P2) is inside of *gidB*, primer 3 (P3) in inside of zeocin resistance marker. See Table S3 for sequences of primers. Red box means successfully deletion of *gidB*. C. RT-qPCR to further verify the *gidB* mRNA level in *gidB* deletion strains and complementation strains.

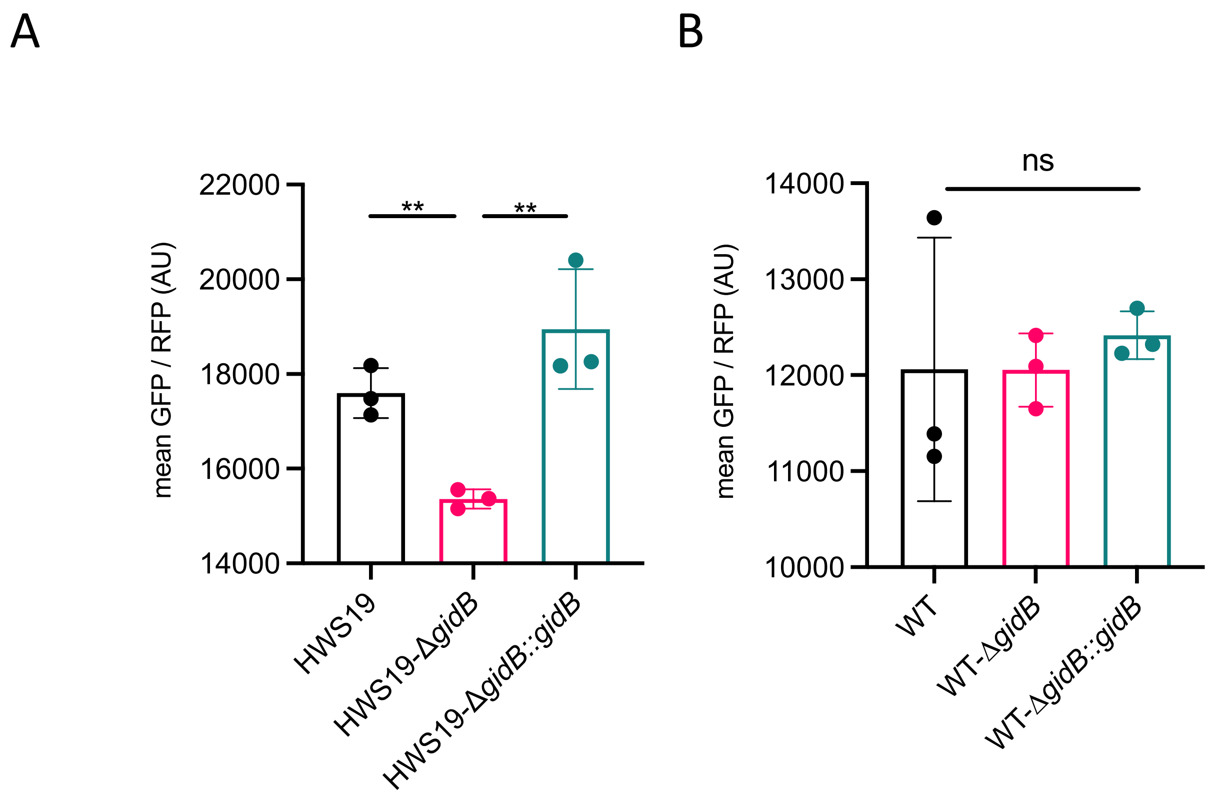

Figure S4. *gidB* deletion increases translation fidelity in high mistranslation mutant.
E to Q mistranslation measured by gain-of-function dual fluorescent reporter (Figure.2B) in high mistranslation HWS19 strain (A) and wild type strain (B)

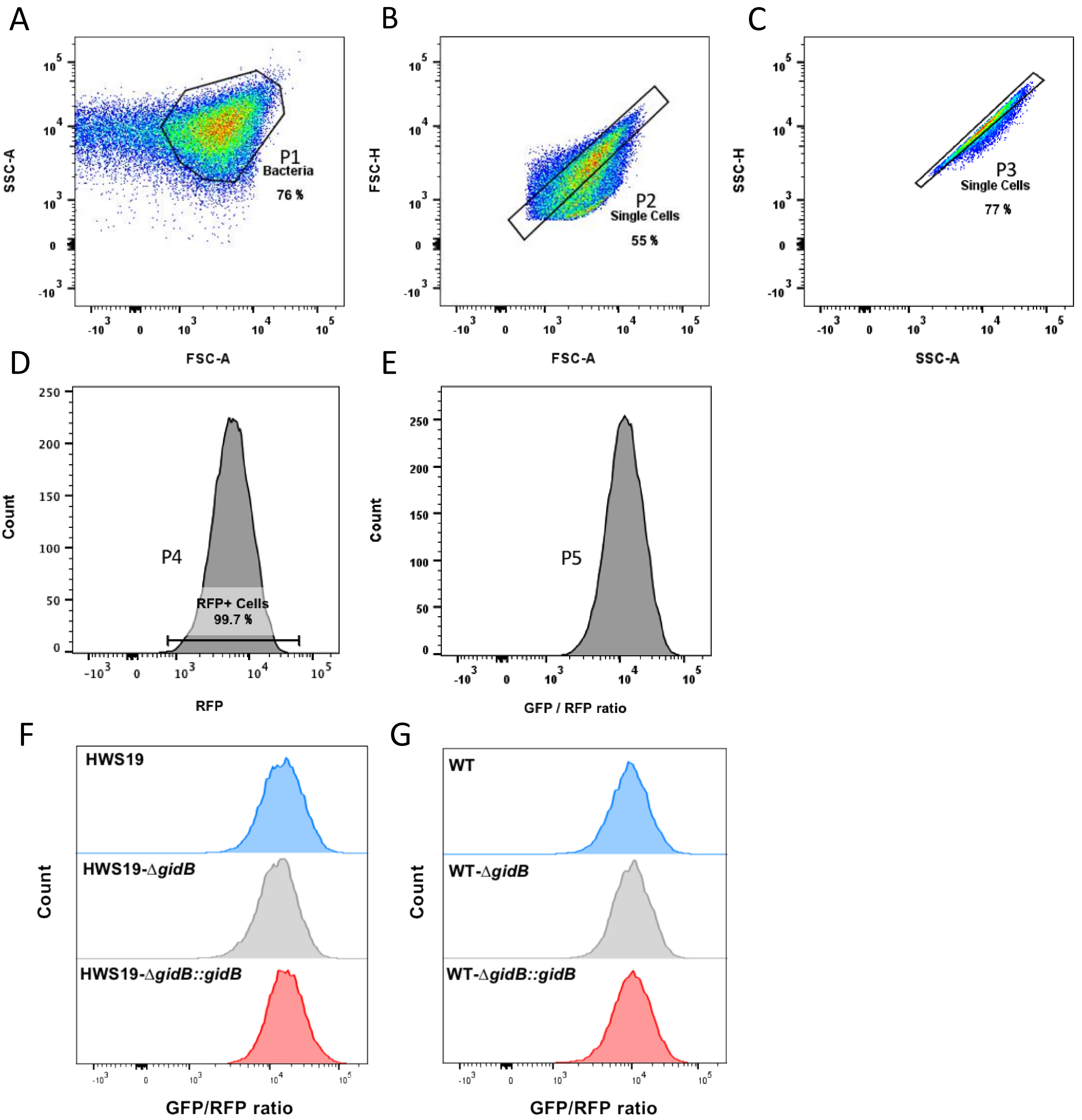

Figure S5. Gating strategy of flow cytometry and histogram of population count replying on GFP/RFP ratio. A. Gating *M.smegmatis* based on the estimation of the size and granularity as P1 in terms of SSC-A (X axis) and FSC-A (Y axis). B. Gating single-cell as P2 in terms of FSC-A (X axis) and FSC-H (Y axis). C. Gating single-cell again as P3 in terms of SSC-A (X axis) and SSC-H (Y axis). D. Gating smegmatis expressing positive RFP as P4 in terms of RFP (X axis). E. Histogram of population count (Y axis) relying on GFP/RFP ratio (X axis). F.G. GidB deletion strains and complementation strains in high mistranslating background (F) and wild-type background (G). X axis is the value of GFP and RFP ratio, representing the Q to E mistranslation level. The strain distributes to the right presents a higher mistranslation level than the one to the left.

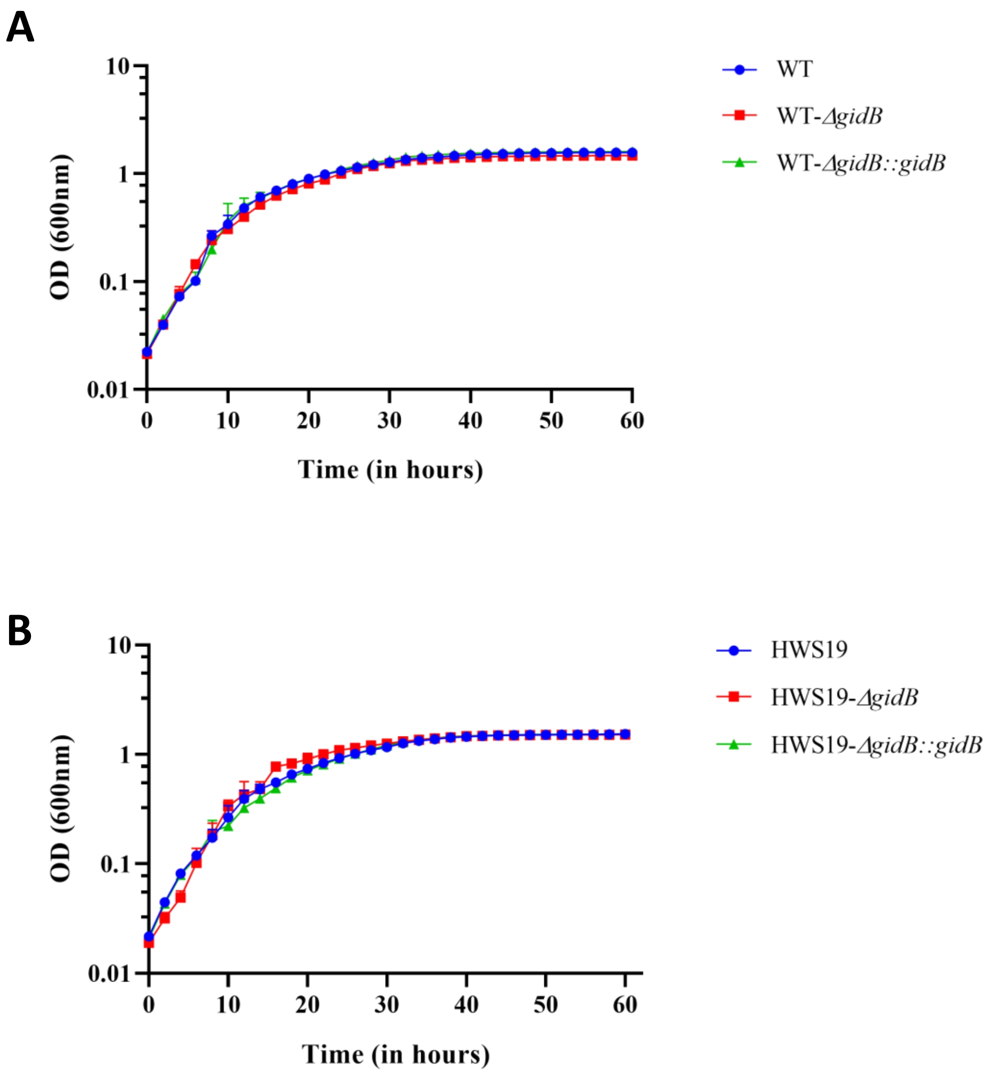

Figure S6. Deletion of GidB does not affect growth rate in axenic culture. WT Msm and Δ*gidB* (A) and strain HWS19 and HWS19Δ*gidB* (B) and their respective complemented strains were grown in standard supplemented 7H9 medium and optical density monitored over time. Points represent means +/- SD.

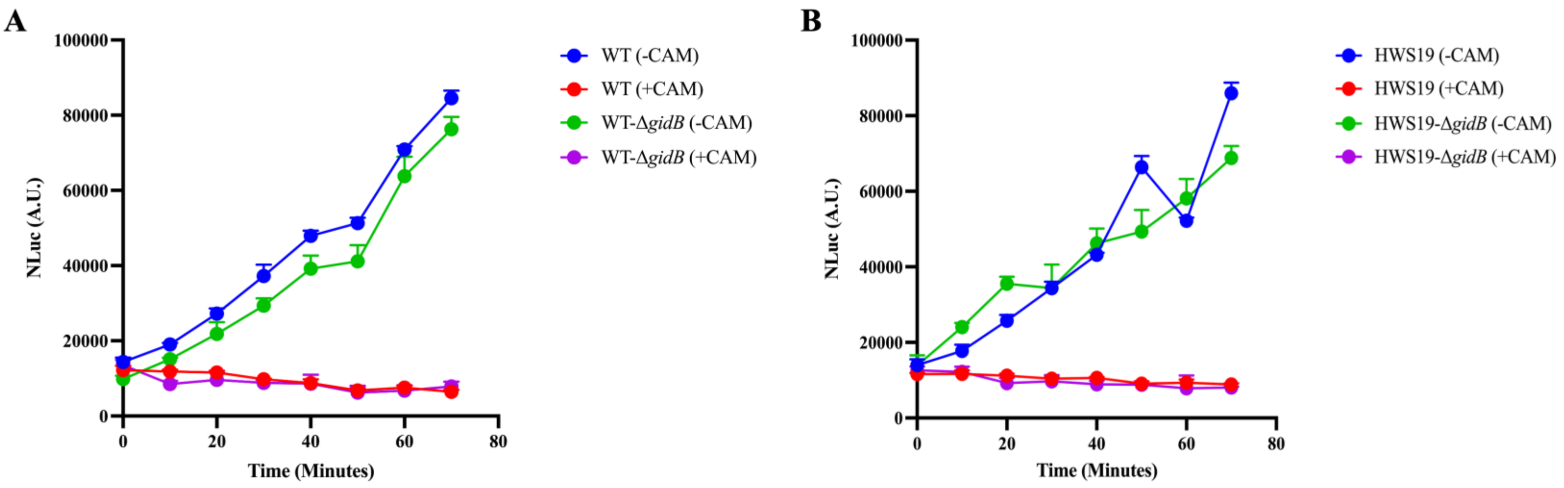

Figure S7. Deletion of GidB does not affect translation rate of Nluc luciferase. WT Msm and Δ*gidB* (A) and strain HWS19 and HWS19Δ*gidB* (B) were transformed with a tetrycline-inducible Nluc luciferase construct. Nluc activity was monitored over time as a proxy for translation rate in all 4 strains. Time =0 represents time of addition of Atc (50ng/mL). Experiment was performed using three independent biological replicates. Points represent means +/- SD. Chloramphenicol (200 µg/mL) was used as a translation inhibitor control.

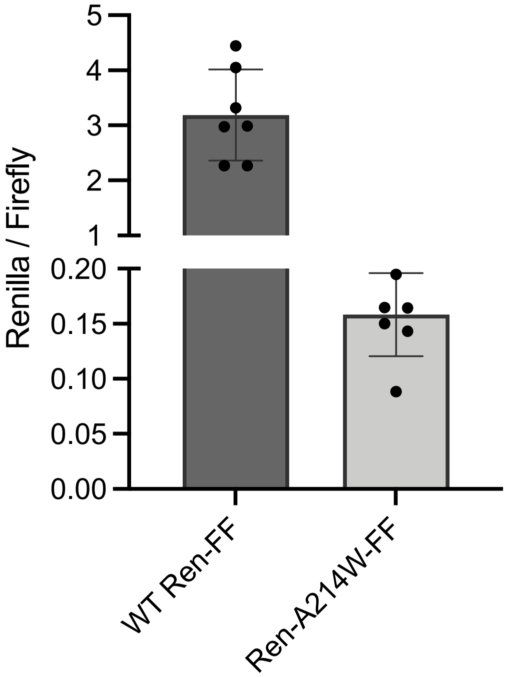

Figure S8. Corrected Renilla luciferase activity. Renilla-WT_Firefly and Renilla-A214W_Firefly were transformed into wildtype smegmatis. The absolute ratio of Renilla-WT/ Firefly and Renilla-A214W/ Firefly were illustrated in the graph.

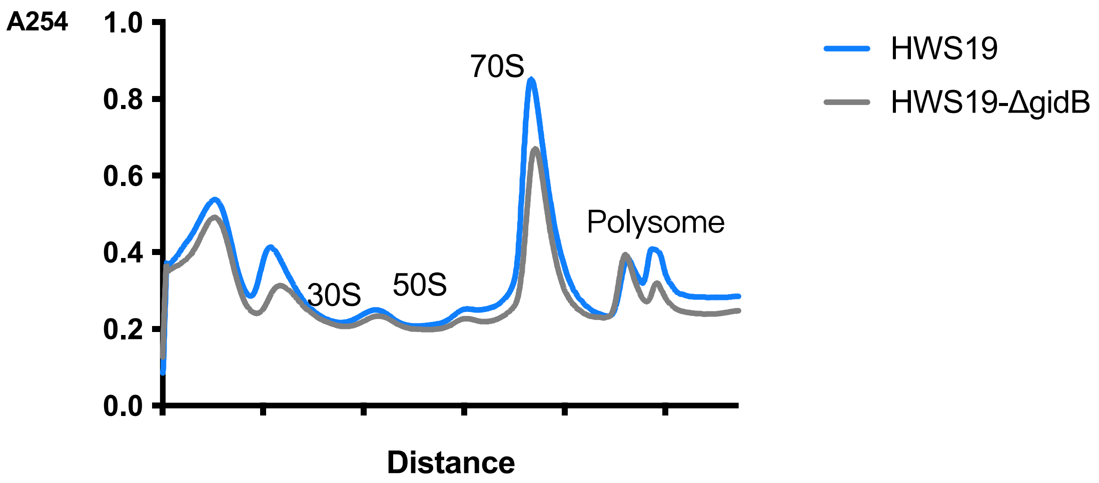

Figure S9. Subunit profiling of different *M.smegmatis* strains. Sucrose gradient profile of HWS19, HWS19- ΔgidB.
